# Supplementary material for: Green Synthesis of CuO Nanoparticles from Macroalgae Ulva lactuca and Gracilaria verrucosa
Source: Nanomaterials (Basel). 2024 Jul 6;14(13):1157. doi: 10.3390/nano14131157 (PMC11243669; doi:10.3390/nano14131157)
Supplement: Supplementary file 1 [file nanomaterials-14-01157-s001.zip › nanomaterials-3077680-supplementary.pdf]

Supplementary Materials

Green Synthesis of CuO Nanoparticles from Macroalgae *Ulva lactuca* and *Gracilaria verrucosa*

Marta Marmiroli [1,*], Marco Villani [2], Paolina Scarponi [1], Silvia Carlo [1], Luca Pagano [3], Valentina Sinisi [2], Laura Lazzarini [2], Milica Pavlicevic [1] and Nelson Marmiroli [3]

[1] Department Chemistry, Life Sciences, and Environmental Sustainability, University of Parma, Parco Area delle Scienze, 43124 Parma, Italy; paolina.scarponi@unipr.it (P.S.); milica.pavlicevic@unipr.it (M.P.)
[2] Istituto dei materiali per l'elettronica ed il magnetismo (CNR IMEM), Parco Area delle Scienze, 43124 Parma, Italy; marco.villani@imem.cnr.it (M.V.); valentina.sinisi@imem.cnr.it (V.S.); laura.lazzarini@imem.cnr.it (L.L.)
[3] Consorzio Interuniversitario Nazionale per le Scienze Ambientali (CINSA), University of Parma, Parco Area delle Scienze, 43124 Parma, Italy; luca.pagano@unipr.it (L.P.); nelson.marmiroli@unipr.it (N.M.)
* Correspondence: marta.marmiroli@unipr.it

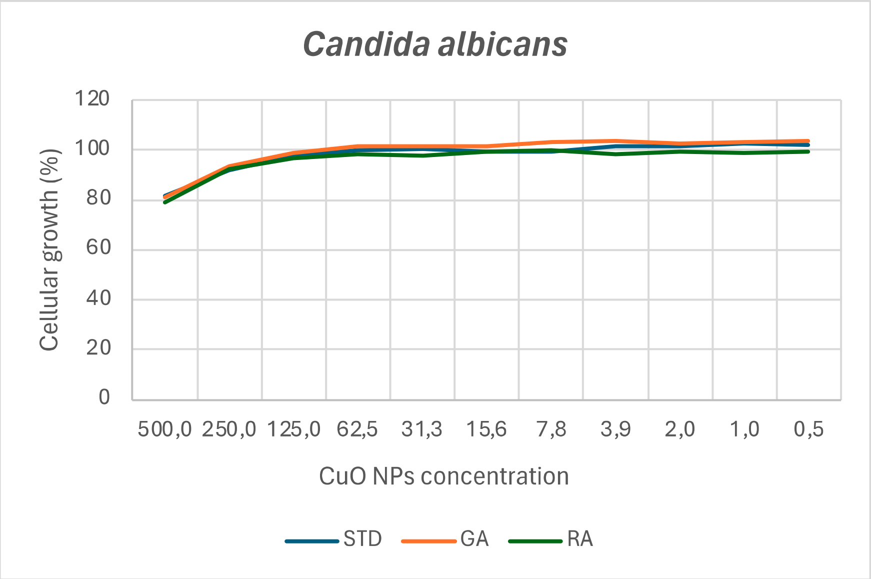
Candida albicans
120
100
80
60
40
20
0
Cellular growth (%)
500,0 250,0 125,0 62,5 31,3 15,6 7,8 3,9 2,0 1,0 0,5
CuO NPs concentration
STD GA RA

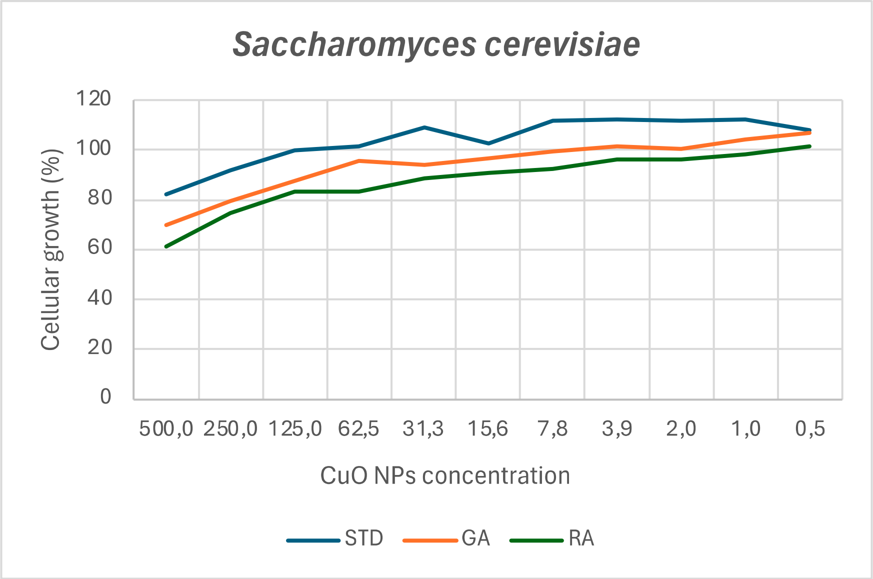
Saccharomyces cerevisiae
120
100
80
60
40
20
0
Cellular growth (%)
500,0 250,0 125,0 62,5 31,3 15,6 7,8 3,9 2,0 1,0 0,5
CuO NPs concentration
STD GA RA

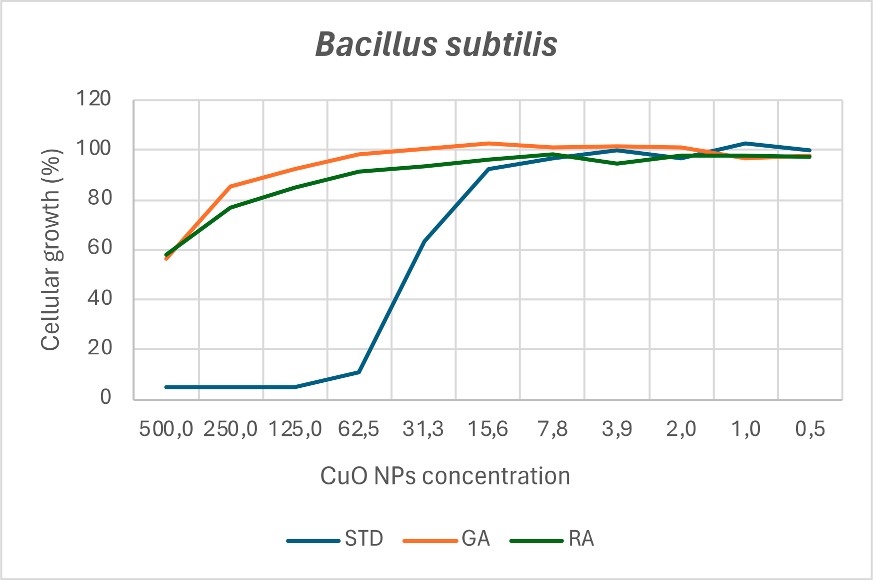
Bacillus subtilis
120
100
80
60
40
20
0
Cellular growth (%)
500,0 250,0 125,0 62,5 31,3 15,6 7,8 3,9 2,0 1,0 0,5
CuO NPs concentration
STD GA RA

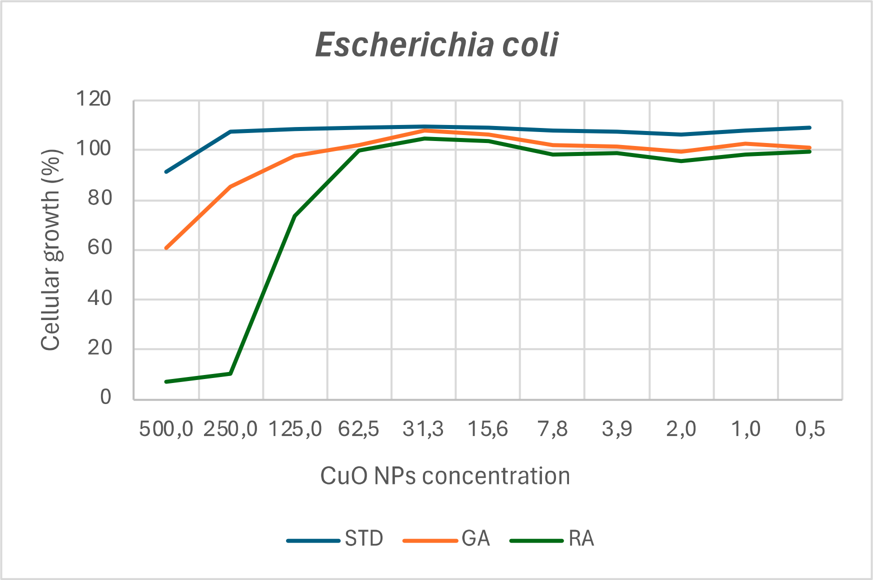

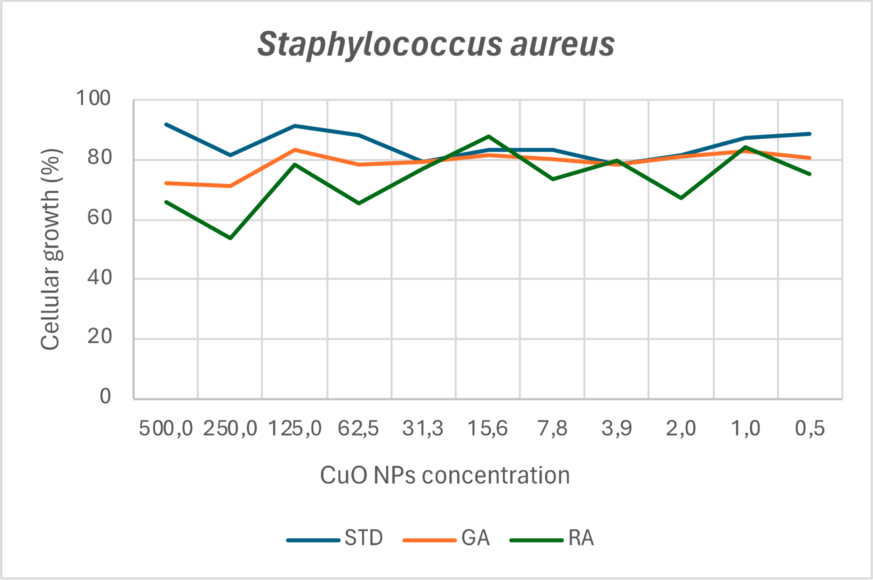

Figure S1. The plots represent the variation of microorganism's cellular growth with decreasing CuO NPs concentrations (mg L^{-1}), which is reported in detail in Table 1. The cellular growth is represented as the growth percentage of CuO NPs treated microorganisms compared to the untreated control, where untreated control cellular growth is 100% and sample cellular growth is calculated as ($OD_{600\ sample}$ * 100)/$OD_{600\ control}$.

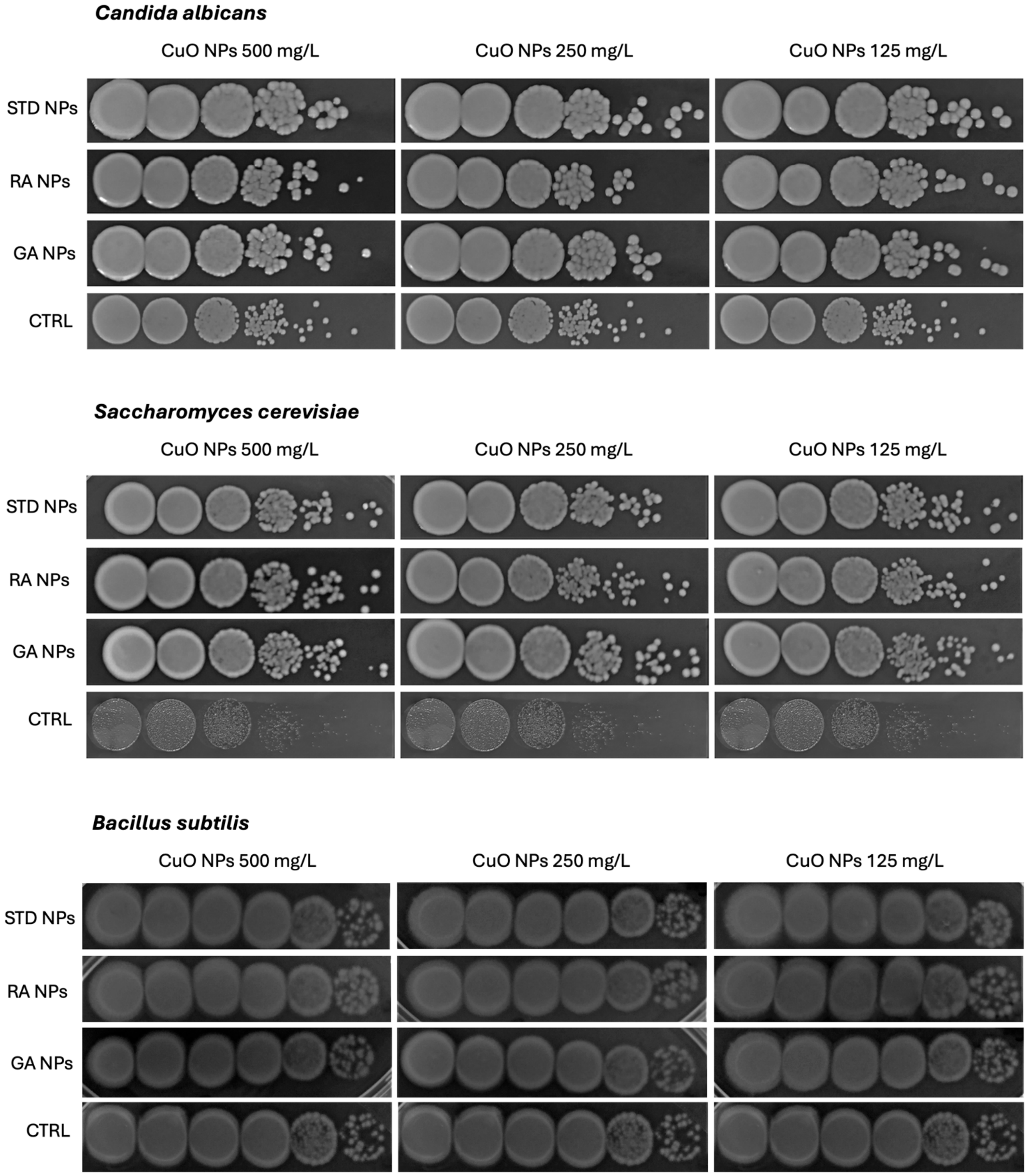
Candida albicans
CuO NPs 500 mg/L
CuO NPs 250 mg/L
CuO NPs 125 mg/L
STD NPs
RA NPs
GA NPs
CTRL
Saccharomyces cerevisiae
CuO NPs 500 mg/L
CuO NPs 250 mg/L
CuO NPs 125 mg/L
STD NPs
RA NPs
GA NPs
CTRL
Bacillus subtilis
CuO NPs 500 mg/L
CuO NPs 250 mg/L
CuO NPs 125 mg/L
STD NPs
RA NPs
GA NPs
CTRL

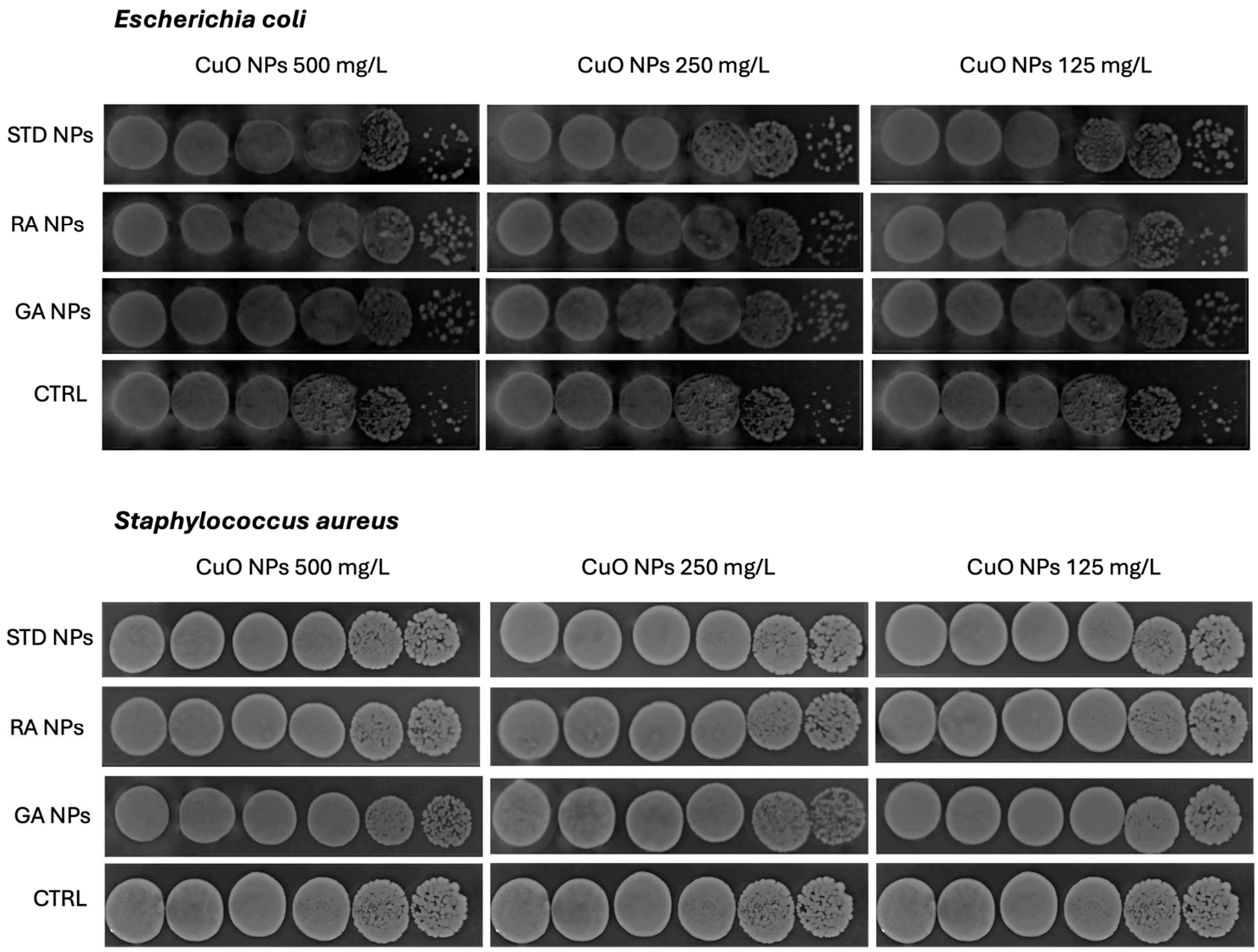

Figure S2. Spot assay of serial dilution of microorganisms treated with CuO NPs on solid media. First concentration (left) represented a concentration of 1 OD, serial dilutions (factor 1:10) have been performed. STD= standard CuO NPs, GA: CuO NPs synthesized from green algae, RA: CuO NPs synthesized from red algae, CTRL: untreated microorganisms (0 mg L^{-1}). 10 μL of cell culture were plated on agar medium after 24 h of incubation at 28 °C with the NPs treatments under analysis.

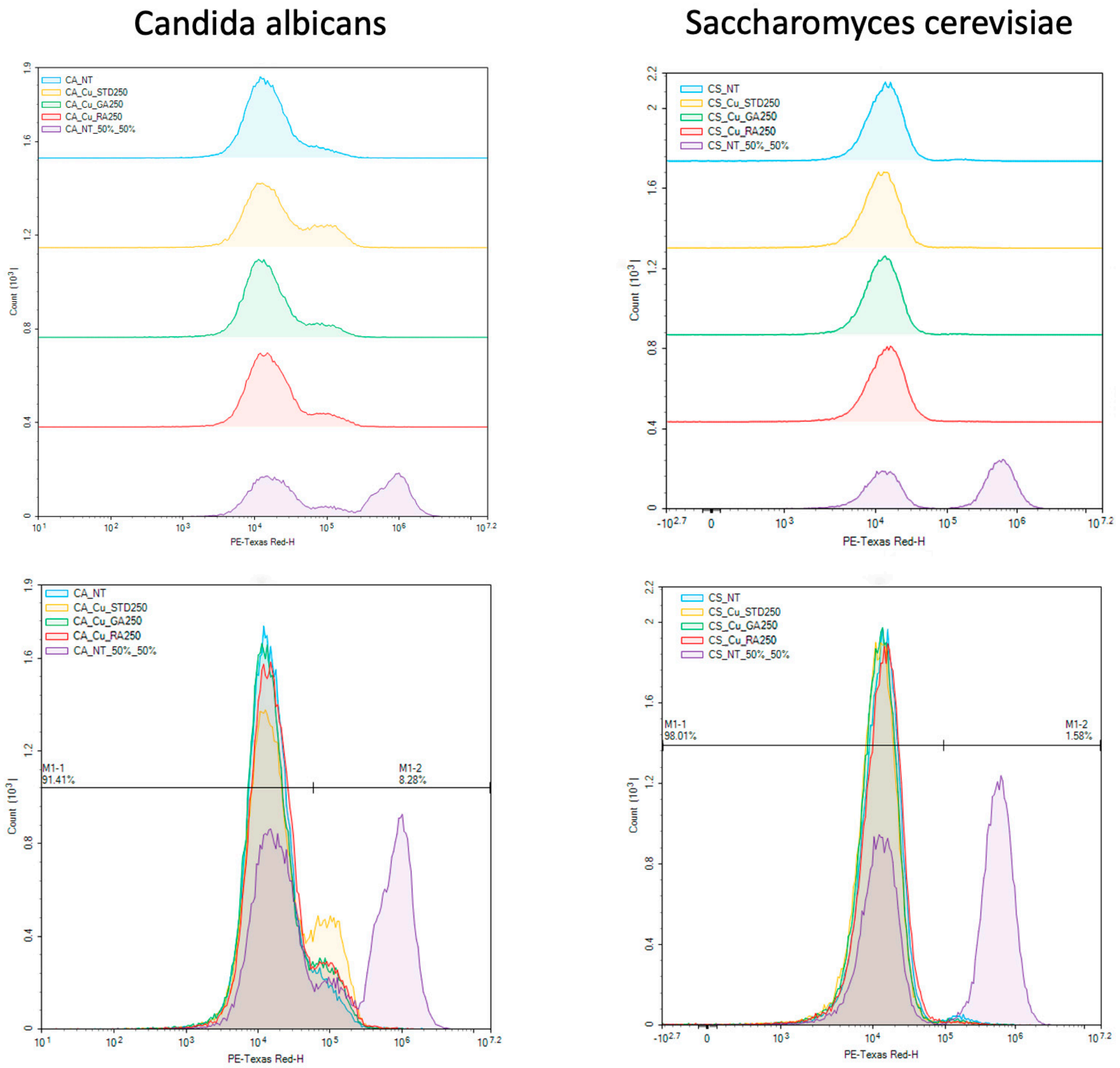

Figure S3. Distribution diagrams of flow cytometer PI signal for cell mortality of *C. albicans* and *S. cerevisiae* treated with 0 and 250 mg L^{-1} of commercial CuO NPs (STD) and CuO NPs biosynthesized for green (GA) and red algae (RA). As an internal standard, a sample with induced 50% cell mortality, has been included

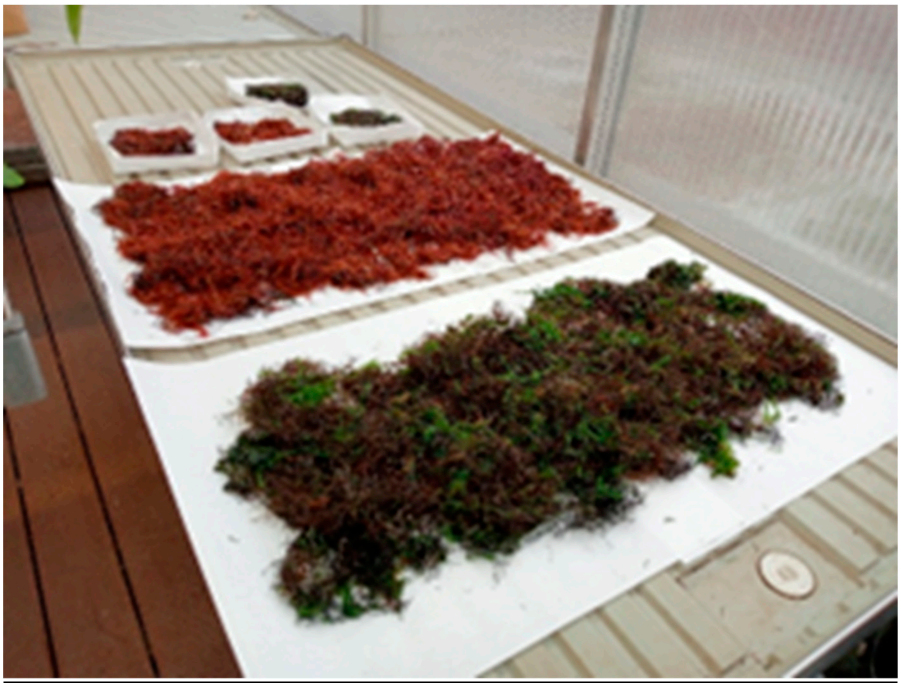

Figure S4. Algae during the desiccation phase

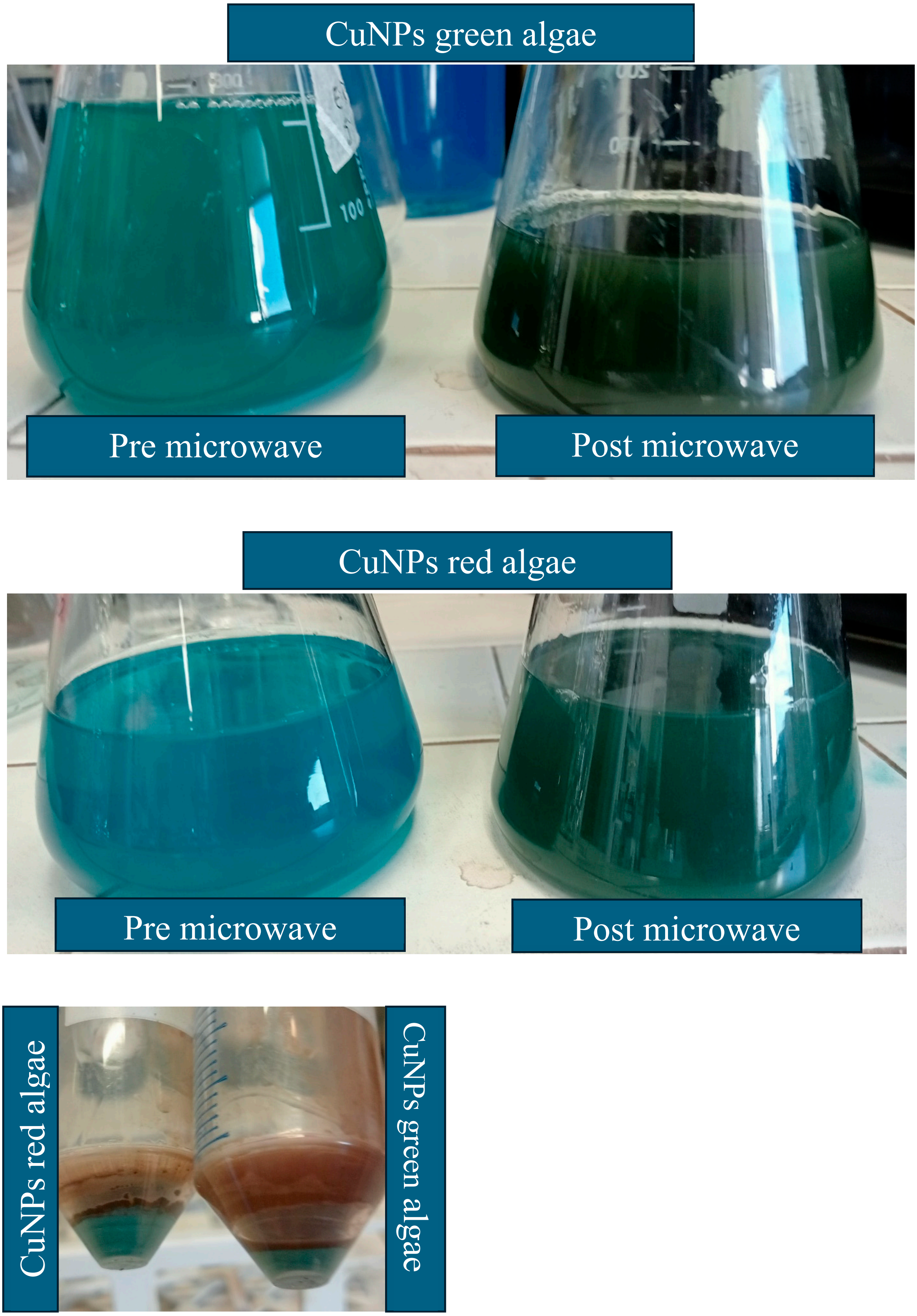

Figure S5. Change in color of the algae extracts before and after microwave treatment.
